# Neuroprotective Activity of Melittin—The Main Component of Bee Venom—Against Oxidative Stress Induced by Aβ_25–35_ in In Vitro and In Vivo Models

**DOI:** 10.3390/antiox10111654

**Published:** 2021-10-21

**Authors:** Cong Duc Nguyen, Gihyun Lee

**Affiliations:** College of Korean Medicine, Dongshin University, Naju 58245, Korea; Ducngcong@dsu.ac.kr

**Keywords:** bee venom, BDNF, beta amyloid, oxidative stress, neurodegeneration, melittin

## Abstract

Melittin, a 26-amino acid peptide, is the main component of the venom of four honeybee species and exhibits neuroprotective actions. However, it is unclear how melittin ameliorates neuronal cells in oxidative stress and how it affects memory impairment in an in vivo model. We evaluated the neuroprotective effect of melittin on Aβ_25–35_-induced neuro-oxidative stress in both in vitro HT22 cells and in vivo animal model. Melittin effectively protected against HT22 cell viability and significantly deregulated the Aβ_25–35_-induced overproduction of intracellular reactive oxygen species. Western blot analysis showed that melittin suppressed cell apoptosis and regulated Bax/Bcl-2 ratio, as well as the expression of proapoptotic related factors: Apoptosis-inducing factor (AIF), Calpain, Cytochrome c (CytoC), Cleaved caspase-3 (Cleacas3). Additionally, melittin enhanced the antioxidant defense pathway by regulating the nuclear translocation of nuclear factor erythroid 2-like 2 (Nrf2) thus upregulated the production of the heme oxygenase-1 (HO-1), a major cellular antioxidant enzyme combating neuronal oxidative stress. Furthermore, melittin treatment activated the Tropomyosin-related kinase receptor B (TrkB)/cAMP Response Element-Binding (CREB)/Brain-derived neurotrophic factor (BDNF), contributing to neuronal neurogenesis, and regulating the normal function of synapses in the brain. In our in vivo experiment, melittin was shown to enhance the depleted learning and memory ability, a novel finding. A mouse model with cognitive deficits induced by Aβ_25–35_ intracerebroventricular injection was used. Melittin had dose-dependently enhanced neural-disrupted animal behavior and enhanced neurogenesis in the dentate gyrus hippocampal region. Further analysis of mouse brain tissue and serum confirmed that melittin enhanced oxidant–antioxidant balance, cholinergic system activity, and intercellular neurotrophic factors regulation, which were all negatively altered by Aβ_25–35_. Our study shows that melittin exerts antioxidant and neuroprotective actions against neural oxidative stress. Melittin can be a potential therapeutic agent for neurodegenerative disorders.

**Highlights**

Melittin alleviates HT22 in vivo and in vitro oxidative stress injury induced by Aβ_25–35_; For the first time, melittin has been reported to enhance cognitive function in a learning memory-deficit model; Melittin ameliorating mechanisms were observed via the nuclear translocation of Nrf2, stimulating 1-HO production and the regulation of the TrkB/CREB/BDNF pathway.

## 1. Introduction

For centuries, bee venom therapy has been used to manage acute and chronic human diseases; recently, this focus has steadily gained more interest from researchers and practitioners for bee venom’s anti-neurodegeneration ability [1]. Melittin, a 26-amino acid peptide, is the dominant component in the venom of four most common honeybee species—*Apis dorsata*, *Apis mellifera*, *Apis florea*, and *Apis cerena*, which contain 95.8 ± 3.2%, 76.5 ± 1.9%, 66.3 ± 8.6%, and 56.8 ± 1.8% melittin, respectively [2,3].

Regarding neuroprotective effects, as evidenced by previous studies, melittin exhibits suppressive effects on the proinflammatory, and potentially pro-oxidative, response of BV2 microglia including the ability to reduce nitric oxide (NO) and inducible nitric oxide synthase (iNOS) by blocking LPS-induced activation of NF-κB7, suppressing the expression of COX-2/PGE2, resulting in anti-inflammatory properties [4]. Another study revealed that melittin stabilized pros and anti-apoptosis factor in SY5Y human neuroblastoma cells under stress induced by H_2_O_2_ [5].

As the primary component of honeybee venoms, melittin potential is high, and more research needs to be conducted on this compound to further explore its role in combating neurodegeneration. Neuro-oxidative stress ignited by beta amyloid (Aβ) in the human brain is a main trigger for the progression of neural disorder [6,7], and melittin’s protective capacity and mechanisms in this aspect are still unclear. Further, at an in-vivo level, the ability of melittin to convalesce deficit cognitive function remains elusive.

In previous cardiology and renal studies, melittin was reported to regulate the nuclear translocation of Nrf2, a key transcription factor that upregulates HO-1 expression [8,9]. HO-1 is a critical cellular antioxidant enzyme that normalizes redox balance in various disorders including in neurodegenerative oxidative stress [10]. A step from this, in an anti-neurodegeneration study, the TrkB/CREB/BDNF loop was also a companionable route that inter-connects and is commonly studied alongside cellular antioxidant aspects [10,11]. Therefore, we hypothesized that melittin also protects neuron cells such as HT22 against neurodegeneration induced by oxidative stress by regulating the Nrf2/HO-1, and TrkB/CREB/BDNF pathways; consequently, this substance has a high chance of exhibiting protection effects in a neurodegenerative cognitive induced in vivo model.

Regarding the establishment of a neurodegenerative model for this study: Aβ aggregated species in neurodegenerative brains is considered a major cause of the onset of cognitive dysfunction disease because of its ability to alter intercellular redox balance [7,12,13]. Among Aβ variances, the Aβ_25__–35_ fragment is the shortest segment that was proven to have similar oxidative neurotoxicity output compared with the full-length Aβ_1__–42_ [14]. With good solubility and stress-induced efficiency [15], in recent decades, Aβ_25–35_ has become a common neurodegeneration igniter agent that simulates in vitro intercellular damage caused by excessive redox impacts [16,17,18,19] and also induces oxidative stress, synaptic loss, suppress neurogenesis, and impairment cognitive function in animal models [15,20,21,22].

## 2. Material and Methods

### 2.1. Materials

We purchased 1 × DMEM, 1 × PBS, and 1 × TBS from Welgene, Inc. (Gyeongsan, Gyeongbuk, Korea). Trypsin-EDTA (0.25%), FBS, 2,7-dichlorofluorescein diacetate (DCFDA), and penicillin–streptomycin were obtained from Invitrogen (Carlsbad, CA, USA). Melittin, Aβ_25–35_, and Fluoromount™ Aqueous Mounting Medium were purchased from Sigma–Aldrich (St. Louis, MO, USA), Aβ_25–35_ aggregation was performed (at pH 7.4, 37 °C for four days) before being introduced to the experiments [23]. The Quanti MAX^TM^ WST-8 Cell Viability Assay Kit was obtained from BIOMAX (Seoul, Korea). The specific mouse primary antibodies (Bax, Bcl-2, TrkB, p-TrkB, CREB, p-CREB, 1-HO, Nrf2, iNOS, and β-actin) and the secondary anti-mouse IgG (HRP-linked antibody) were purchased from Cell Signaling Technology, Inc. (Danvers, MA, USA). Specific BDNF, lamin B, and mAChR1 antibodies were purchased from Merck KGaA (Darmstadt, Germany). Pro-PREP protein extraction solution was purchased from iNtRON Biotechnology (Seoul, Korea). First doublecortin antibody (sc-271390, Santa Cruz Biotechnology, CA, USA) and Alexa Fluor 488 secondary antibody (Thermo Fisher Scientific, Waltham, MA, USA) were used. Cellular Reactive oxygen species (ROS), SOD (Superoxide Dismutase), Lipid Peroxidation (MDA), Lactate Dehydrogenase (LDH), and protein carbonyl activity required acetylcholine esterase (AchE) assay kits, AIF first antibody, CytoC first antibody, and CleaCas3 first antibody (Abcam, Cambridge, UK). A nuclear extraction kit was purchased from Cayman Chemical Company, Inc. (Ann Arbor, MI, USA). An Ach level assay kit was obtained (Cell Biolabs, San Diego, CA, USA).

### 2.2. Cell Culturing

The HT22 mouse hippocampal cell line was kindly gifted by Professor Na Chang Su and cultured in Dulbecco’s modified Eagle’s medium (DMEM) supplemented with FBS (10%) and penicillin–streptomycin (1%) in an incubator at 5% CO_2_ and 37 °C. Cells were cultured at a density of 1 × 10^4^ cells per well (with 0.1 mL media) in a 96-well plate, and 3 × 10^5^ cells per well (with 3 mL media) in a 6-well plate. Twenty-four hours after seeding, drugs were administrated into the wells as explain in the next parts.

### 2.3. Cell Viability Assay and Drug Administration

To assess cell viability in 96-well plates, after 24 h, melittin was added to the wells (0.1 to 30 μM for a safety concentration test and 0.1 to 3 μM for cellular protection activity), and 1 h later, 7 μM of Aβ_25–35_ was added. Cells were incubated for another 24 h, followed by incubation with WST-8 for 1 h. Absorbance was measured at 450 nm using a VersaMax Microplate Reader (Molecular Devices, San Jose, CA, USA). The experiments were repeated three times.

### 2.4. Measurements of In Vitro Cell Apoptosis, Cell ROS, SOD, MDA, LDH, and Protein Carbonyl

For apoptosis and ROS immunofluorescent experiments: In black 96-well, clear bottom plates, experiment started 7 h after treatment with Aβ_25–35_. For apoptosis assay, cells were washed with kit assay buffer twice, then to each well we added 200 µL assay buffer + 1 µL kit solution of CytoCalcein 450 (for live cell detection) + 1 µL kit solution of 7-AAD (for detecting necrotic cells) + 2 µL kit solution of Apopxin Green Indicator (for detecting apoptosis cells), followed by incubated at room temperature, in the dark, for 45 min. Subsequently, cells were washed once and replaced with 100 µL assay buffer. Live cells were detected using a 4′,6-diamidino-2-phenylindole (DAPI) channel (Ex/Em = 405/450 nm), necrotic cells were detected using a Red Fluorescent Protein (RFP) channel (Ex/Em = 550/650 nm), and apoptosis cells were detected using a Green Fluorescent Protein (GFP) channel (Ex/Em = 490/525 nm) with a 30× objective. In total, three images were taken at the center of three replicated wells (containing a sum of 270–330 cells), and the apoptosis cell percentage was calculated relative to the total number of observed cells in each image. For ROS assay, cells were washed with kit assay buffer twice, and then each well was filled with 200 µL of 25 μM DCFDA solution and incubated at 37 °C, in the dark, for 45 min. The DCFDA solution was then removed and replaced with 100 µL assay buffer. ROS fluorescence levels were detected via the GFP channel (Ex/Em = 490/525 nm) with a 30× objective. In total, three images were taken at the center of three replicate wells and analyzed with ImageJ software (National Institutes of Health, Bethesda, MD, USA). An Invitrogen EVOS FL Auto Imaging System (Thermo Fisher scientific, Waltham, MA, USA) fluorescence microscope was used.

For SOD, MDA, LDH, and protein carbonyl assays: In 6-well plates, the experiment started 7 h after treatment with Aβ_25–35_; cell lysates were performed as per the kit instructions, with SOD, MDA, LDH, and protein carbonyl levels according to the manufacturer’s instructions. The data were collected using a VersaMax Microplate Reader (Molecular Devices, San Jose, CA, USA).

### 2.5. Preparation of Whole Cell, Cytosolic, and Nuclear Proteins

To examine the nuclear translocation of Nrf2, we fractionated nuclear and cytosolic proteins from the whole cell content [11]. Cells were grown in a 6-well plate, and after treatment with Aβ_25–35_ for 7 h, cells were harvested. A part of the cell pellet was lysed to obtain whole-cell protein. The other part was mixed with hypotonic buffer containing phosphatase and protease inhibitors. After 10 min of incubation at 4 °C, 10% Nonidet P-40 Assay Reagent was added to stimulate phase separation. After centrifugation (14,000× *g*, 30 s), the supernatant as the cytosolic extract was stored at −80 °C until use. The remaining pellets were added to the nuclear extraction buffer for 30 min at 4 °C. After centrifugation (14,000× *g*, 10 min), the supernatant was collected as the nuclear extract.

### 2.6. Animals

ICR mice (males; age: 6 weeks; body weight: 25–30 g) were obtained from Dehan Biolink Co. (Eumseong, Korea) and housed with two mice per cage, with specific pathogen-free conditions (temperature: 22–26 °C; relative humidity: 50–60%) under a 12-h light/dark cycle and with free access to standard mouse food (Sangyang Co., Osen, Korea) and water. The mice were acclimatized for five days before the experiments were initiated. All behavioral experiments were conducted under the same ambient conditions according to the Guide for Care and Use of Laboratory Animals of the National Research Council (NRC, 1996) and were approved by the Committee of Animal Care and Experiment of Dongshin University, Korea (DSU2019-04-02).

### 2.7. In Vivo Drug Administration

Aβ_25–35_ intracerebroventricular (ICV.) injections were performed as described in previous studies: The prepared Aβ_25–35_ solution or PBS was injected (5 µL) into the right cerebral ventricle using a 28-gauge stainless steel needle via stereotaxic coordinates (in mm) from the bregma—A: -0.22, L: 1.0, V: 2.5, with flow rate at 5 μL/min [21,24]. On day 1, except for the naïve group which received PBS ICV., all mice were injected with 5 µL PBS solution containing 5 μg of Aβ_25–35_. A melittin maximum safety dosage of approximately 1.5 mg/kg was previously determined [25,26,27]. Therefore, we used 1.5 mg/kg melittin, and another dosage, 0.15 mg/kg melittin, was to study any possible dosage-dependent effect. Melittin was administered via subcutaneous injection (SC.) injections on days 3, 5, 7, 9, and 11. Mice were categorized into the following groups according to treatments: naïve group, PBS ICV. + PBS SC.; control group, 5 μg Aβ_25–35_ ICV. + PBS SC.; Mel 1.5 group, 5 μg Aβ_25–35_ ICV. + melittin 1.5 mg/kg SC.; and Mel 0.15 group, 5 μg Aβ_25–35_ ICV. + melittin 0.15 mg/kg SC. 

### 2.8. Morris Water Maze

The Morris water maze (MWM) test was applied to evaluate the effects of melittin on mice spatial learning and memory as previously described with minor modifications [28]. The MWM equipment consisted of a circular black water tank (diameter: 120 cm; height: 50 cm) surrounded by various visual cues (white star, square, rectangle, and circle one for each). Water temperature was controlled at 22 ± 2 °C. The tank was virtually separated into four equal quadrants: the southeast, northeast, southwest, and northwest quadrants. The platform (diameter: 10 cm; height: 25 cm) was centered in the northwest quadrant. The entire experimental procedure included adaptive training (day 6, three times a day), hidden platform tests (days 7–10, two trials per day), and a spatial probe trial (immediately after the last hidden platform test on day 10, once, 2 min each). The ANY-maze animal behavior monitoring software (Stoelting Co., Wood Dale, IL, USA) was utilized in this experiment.

### 2.9. Collection of In Vivo Animal Tissues

On day 11, all mice were anesthetized, blood and brains samples were collected. In each group, the brains of a subset of animals were used for immunofluorescence analysis (these mice were perused with 4% paraformaldehyde, and postfixed at 4 °C after collection) and the others brain’s hippocampus were collected immediately for biochemical or western blot analysis. 

### 2.10. Doublecortin Immunofluorescence Staining

Brains were post-fixed overnight before being incubated with 30% sucrose in sodium phosphate buffer (0.1 M, pH 7.2) for 24 h at 4 °C. The brains were then frozen by dry CO_2_ ice powder and cut into 25-μm sagittal sections using a cryostat and transferred to gelatin-coated slides [29]. At room temperature, the slides were then incubated with doublecortin primary antibody (1:100, 2 h). After rinsing in PBS twice 30 min each, Alexa Fluor 488 secondary antibody (1:200, 2 h) was applied, followed by two rinses in PBS for 1 h each. Samples were submerged in Fluoromount™ Aqueous Mounting Medium and covered with glass coverslips. The data were collected using an Invitrogen EVOS FL Auto Imaging System (Thermo Fisher Scientific, Waltham, MA, USA) via the GFP channel (Ex/Em = 490/525 nm) with a 30× objective. Numbers of doublecortin positive cells were counted in three slides/animal and three animals/group.

### 2.11. Western Blot Analysis

Cells were grown in 6-well plates. After 7 h of Aβ_25–35_ treatment, the cell pellet was collected and lysed. Mice hippocampus samples were homogenized with lysate buffer immediately after collection. Western blotting was performed as described previously [30]. Proteins transferred onto the membranes were visualized using the WEST One western blot detection system (iNtRON Biotechnology, Inc., Gyeonggi-do, Korea). A protein ladder was used (Thermo Fisher Scientific, Waltham, MA, USA). With ImageJ software (National Institutes of Health, Bethesda, MD, USA), the protein band images were first processed into a binary version (command directory: Process/Binary/Make Binary) and then analyzed to collect signal intensities.

### 2.12. Measurements of ROS, NO, MDA, AchE, and Ach Levels in In Vivo Samples

Hippocampus lysate, or blood serum samples were prepared according to the manufacturer’s instructions. After adjusting the concentrations to normalize total protein levels, ROS, NO, MDA, AchE, and Ach assays were carried out according to the manufacturer’s instructions. Data were collected using a VersaMax Microplate Reader (Molecular Devices, San Jose, CA, USA).

### 2.13. Statistical Analysis

Statistical evaluation was performed using SPSS software, version 18.0, and the data are expressed as the mean ± standard deviation. The presented escape latency and path length data in the MWM test were analyzed by repeated-measures two-way analysis of variance (ANOVA). The other behavioral data and biomarker changes in vitro were tested using one-way ANOVA for multiple comparisons.

## 3. Results

### 3.1. Protective Effect of Melittin on Oxidative Stress-Induced Apoptosis in HT-22 Cells

**Figure 1:**
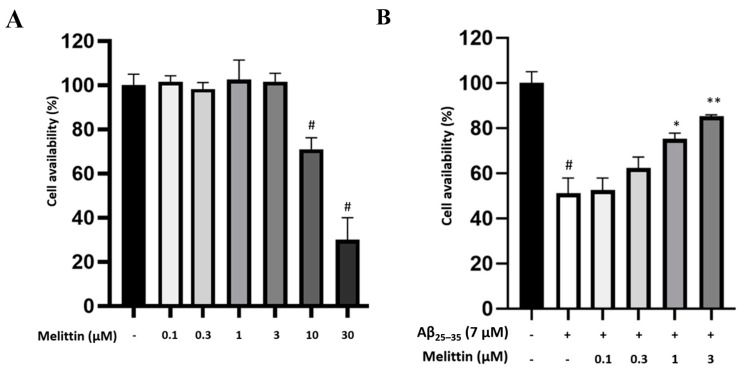
Screening for melittin maximum safety dosage and melittin protective effect against Aβ_25–35_ stress induced in HT22 cells. (**A**) Screening for maximum melittin safety concentration. The result indicated that 3 μM was the highest safety concentration of melittin to be studied. To conduct this experiment: Cells were seeded in 96-well plates; after incubation for 24 h, Melittin at different concentrations was introduced, and cell availability was examined after 24 h of incubation. (**B**) Protective effect of melittin against Aβ_25–35_ stress. The result indicated that 0.3 to 3 μM melittin was found to dosage-dependently ameliorate HT22 cell availability. To conduct this experiment: Cells were seeded in 96-well plates; after incubation for 24 h, melittin from 0.1 to 3 μM was introduced 1 h before Aβ_25–35_ (7 μM) challenge, and cell availability was examined after 24 h of incubation. Data are presented as the mean ± standard deviation values of triple determinations. # *p* < 0.01 vs. control * *p* < 0.05 and ** *p* < 0.01 vs. Aβ_25–35_ only-treated group.

To determine the therapeutic window, we first identified the maximum safety concentrations; we tested melittin concentrations of 0.1–30 μM on HT22 cells and found that a concentration of 3 μM was the maximum safe concentration for our experiment (Figure 1A). Next, for protection effect against 7-μM Aβ_25–35_-induced stress, melittin (0.3–3 μM) exhibited observable dose-dependent effects; therefore, these concentrations were selected for further study (Figure 1B).

**Figure 2:**
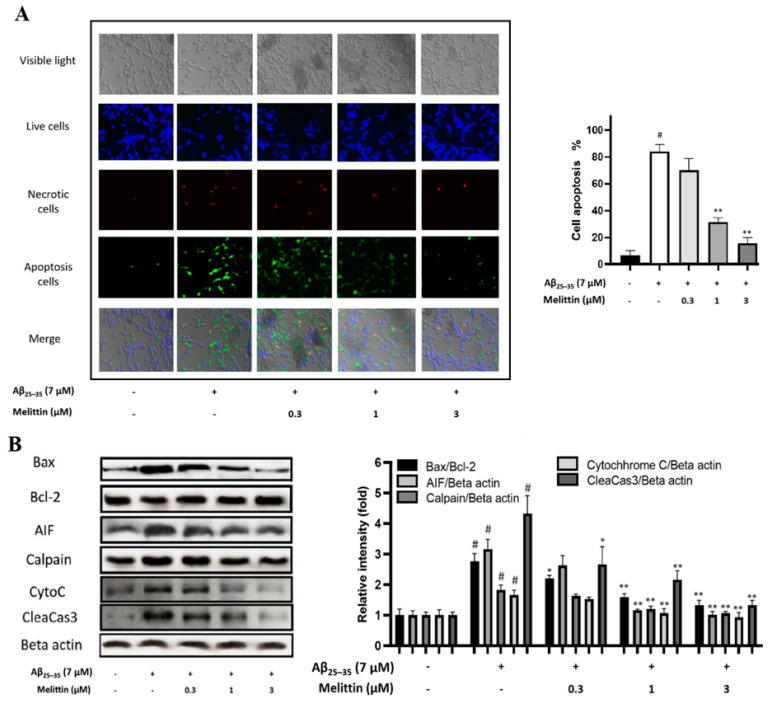
Apoptosis-inhibitory effects of melittin on Aβ_25–35_ induced HT-22 cells. (**A**) Immunofluorescence analysis to determine the cellular apoptosis rate. The result indicated that melittin at 0.3 to 3 μM dosage-dependently ameliorated HT22 cell apoptosis under Aβ_25–35_ (7 μM) stress-induced conditions. To conduct this experiment: Seven hours after Aβ_25–35_ (7 μM) challenge in 96-well plates, immunofluorescence staining was carried out to exhibit live cells (blue, stained by CytoCalcein Violet 450), necrotic cells (red, indicated by 7-AAD staining), and apoptotic cells (green, Apopxin Green Indicator). (**B**) Western blot analysis of key apoptosis proteins. The result indicated that melittin at 0.3 to 3 μM dosage-dependently normalized the expression of pro and anti-apoptosis protein under Aβ_25–35_ stress challenge. To conduct this experiment: Seven hours after Aβ_25–35_ (7 μM) challenge in 6-well plates, cells were lysed, and western blot was performed. Data are presented as mean ± standard deviation values of triple determinations. # *p* < 0.01 vs. control * *p* < 0.05 and ** *p* < 0.01 vs. Aβ_25–35_ only-treated group.

Further experiments were conducted to elucidate the protective effect of melittin against HT22 cell apoptosis. Results revealed that 0.1 μM melittin did not exhibit statistically significant efficacy; however, at 1 and 3 μM, melittin markedly reduced the apoptosis level in HT22 cells by 3- and 4-fold, respectively (Figure 2A). Western blot results indicated that the Bax/Bcl-2 protein ratio was increased 2.5-fold once cells were introduced with Aβ_25–35_, this parameter was dosage dependently normalized by melittin. A step from that, other apoptosis-closely-associated proteins such as AIF, Calpain, CytoC, and CleaCas3 levels were all simultaneously normalized by melittin (Figure 2B) [11,31]. These results are indicators confirming the effect of melittin in protecting HT22 cells against Aβ_25–35_ stress-induced neuronal apoptosis.

### 3.2. Effects of Melittin on Aβ_25–35_-Induced Oxidative Stress

**Figure 3:**
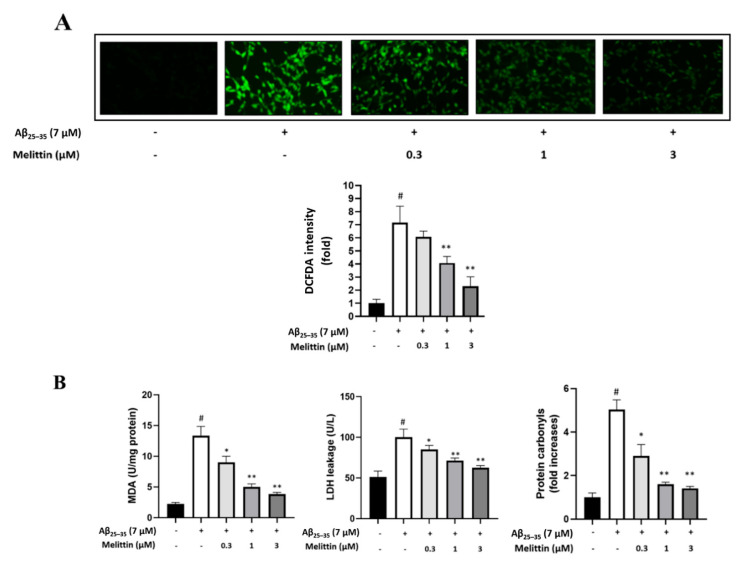
Melittin regulates cellular oxidative stress induced by Aβ_25–35_ in HT22 cells. (**A**) Immunofluorescence analysis to determine the cellular ROS rate. The result indicated that melittin at 0.3 to 3 μM dosage-dependently normalized the expression of pro and anti-apoptosis proteins under Aβ_25–35_ stress challenge. To conduct this experiment: Seven hours after Aβ_25–35_ (7 μM) challenge in 96-well plates, immunofluorescence analysis by DCFDA staining was carried out. (**B**) Cellular MDA, LDH, and protein carbonyls levels. The result indicated that melittin at 0.3 to 3 μM dosage-dependently down-regulated MDA, LDH, and protein carbonyl parameters under Aβ_25–35_ stress challenge. To conduct this experiment: Seven hours after Aβ_25–35_ (7 μM) challenge in 6-well plates, kits measuring MDA, LDH, and protein carbonyls were used to determine the parameters. Data are presented as mean ± standard deviation values of triple determinations. # *p* < 0.01 vs. control * *p* < 0.05 and ** *p* < 0.01 vs. Aβ_25–35_ only-treated group.

Melittin has previously been reported to possess antioxidant activity [2,8,9]. This suggests that the antioxidant properties of melittin can reduce the accumulation of intracellular ROS under Aβ_25–35_ oxidative stress-induced conditions [32]. We found that melittin, 1 and 3 μM, significantly reduced the cellular ROS levels (Figure 3A) by 2- and 3-fold, respectively. This further confirmed that 0.3 to 3 μM melittin dosage-dependently ameliorated the leakage of the key oxidative stress markers MDA and LDH. These excessive destructive cellular free radical sources can modify structure scaffolds and functional apparatus, which eventually increase protein carbonylation [33]. Protein carbonyl is a specific oxidative stress marker that is associated with various disease, including Alzheimer’s disease [34]. Our result indicated that 0.3 to 3 μM melittin exhibited a significant effect in down-regulating cellular protein carbonyl levels (Figure 3B). 

### 3.3. Effects of Melittin on Nuclear Translocation of Nrf2 and Expression of HO-1

**Figure 4:**
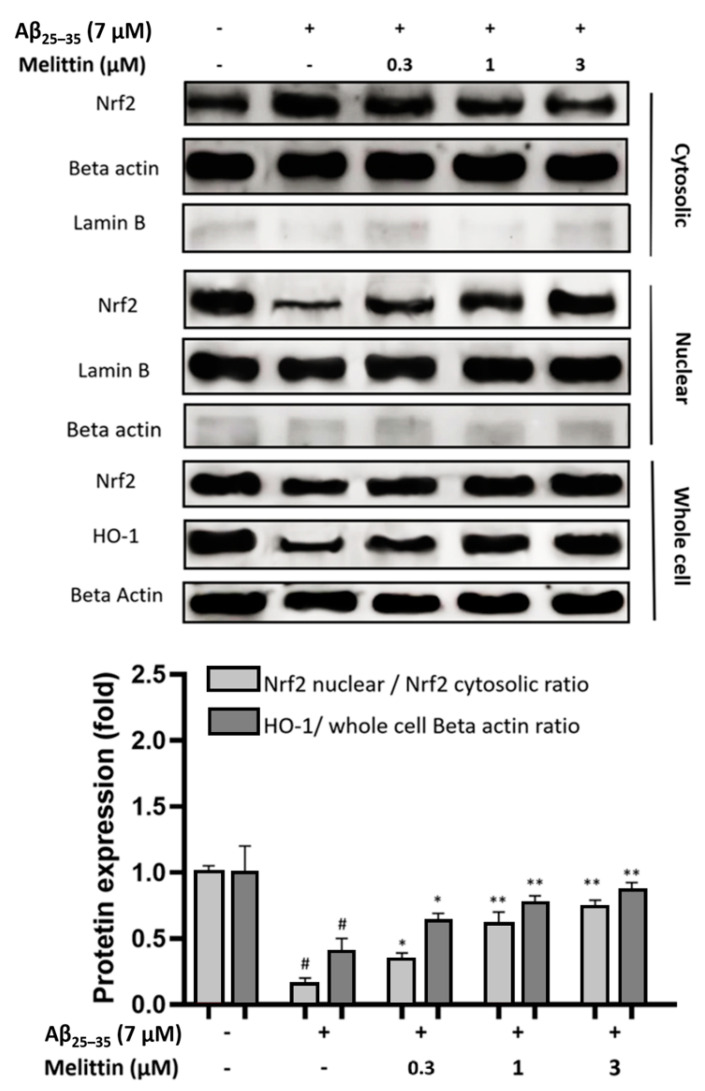
Effect of melittin on nuclear translocation of Nrf2 and an increase in the production of the antioxidant enzyme HO-1. The result suggested that melittin at 0.3 to 3 μM dosage-dependently enhanced Nrf2 nuclear translocation and as a result increased the expression of the HO-1 antioxidant enzyme in HT22 cells under Aβ_25–35_ (7 μM) challenge. To conduct this experiment: Seven hours after Aβ_25–35_ (7 μM) challenge in 6-well plates, whole cell, cytosolic, and nuclear proteins were obtained, and western blot was conducted. The membranes of Nrf2, Lamin B, and Beta actin in cytosolic extract and the membranes of their respective counterparts in nuclear extract under similar treatments and imaging exposure time to express the true scale of protein expression between nuclear and cytosolic proteins. Total Lamin B signal of the cytosolic extract was less than 5% of that in the nuclear extract, total Beta actin in the cytosolic extract was less than 5% of that in the nuclear extract (numeric bars not shown); this indicates the reliability of the manual extraction conducted. Data are presented as mean ± standard deviation values of triple determinations. # *p* < 0.01 vs. Control * *p* < 0.05 and ** *p* < 0.01 vs. Aβ_25–35_ only-treated group.

To further explain the mechanisms by which melittin protects HT22 cells against oxidative stress induced by Aβ_25–35_, we analyzed the improvement in the Nrf2/1-HO pathway under melittin pretreatment. The results indicated that Aβ_25–35_ treatment significantly depleted Nrf2 migration into the cellular nucleus, resulting in the deregulation of the production of the 1-HO antioxidant enzyme. In contrast, melittin (0.3 to 3 μM) pretreatment dose-dependently enhanced Nrf2 nuclear translocation. At 3 μM, melittin treatment markedly increased Nrf2 nuclear translocation by up to 4-fold and HO-1 production by 2-fold compared to Aβ_25–35_ treatment only (Figure 4).

### 3.4. Effect of Melittin on the TrkB/CREB/BDNF Pathway

**Figure 5:**
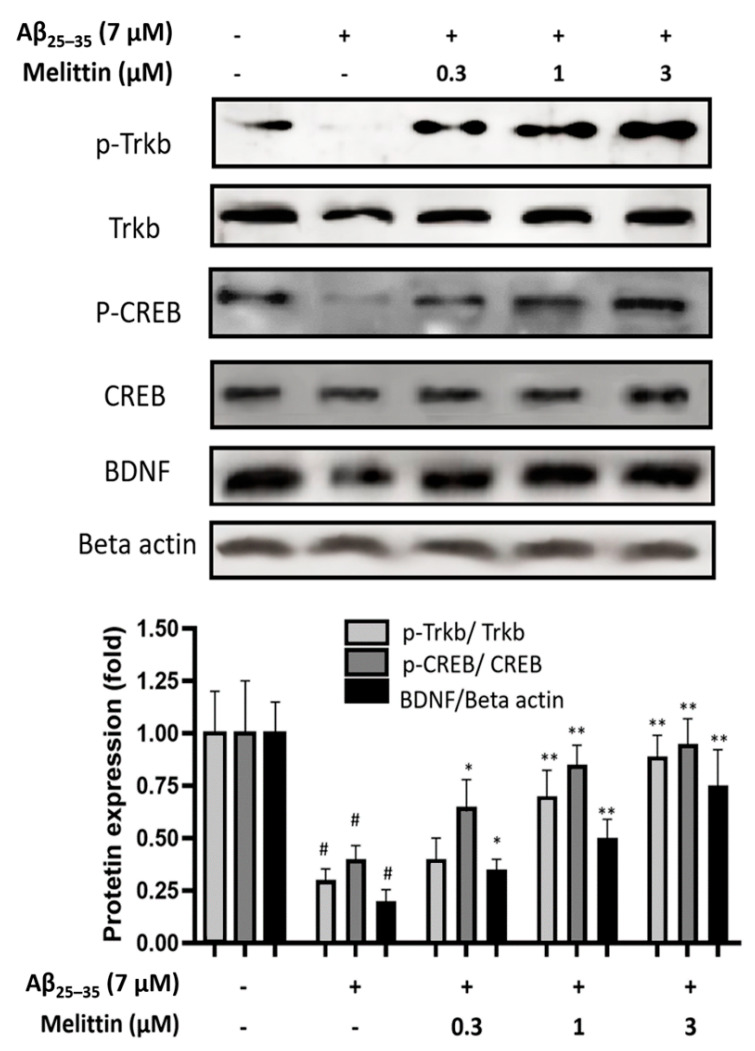
Effects of melittin on the activation of brain-derived neurotrophic factor signaling. The result suggested that melittin enhanced the performance of the BDNF/TrkB/CREB pathway under Aβ_25–35_ (7 μM) challenge. To conduct this experiment: Seven hours after Aβ_25–35_ (7 μM) challenge, cells were lysed, and western blot was performed. Data are presented as mean ± standard deviation values of triple determinations. # *p* < 0.01, vs. control * *p* < 0.05, ** *p* < 0.01, vs. Aβ_25–35_ only-treated group.

In neuroprotective pathways, in addition to Nrf2/HO-1 regulation, TrkB/CREB/BDNF is also known as the key regulator pathway in maintaining neurogenesis [10,11,31]. Our results indicated that melittin plays a role in the stabilization of the TrkB/CREB/BDNF signaling pathway (Figure 5). Melittin dosage-dependent treatment significantly recovered the phosphorylation of p-TrkB, p-CREB, and BDNF expression compared to Aβ_25–35_ treatment only. Taken together, our results showed that melittin exhibited a potent capability in the neuroprotection of HT22 cells under Aβ_25–35_ stress induced via the Nrf2/HO-1 and TrkB/CREB/BDNF pathways.

### 3.5. Improving the Effect of Melittin on Aβ_25–35_ Memory Impairment in Mice in a Water Maze Trial

**Figure 6:**
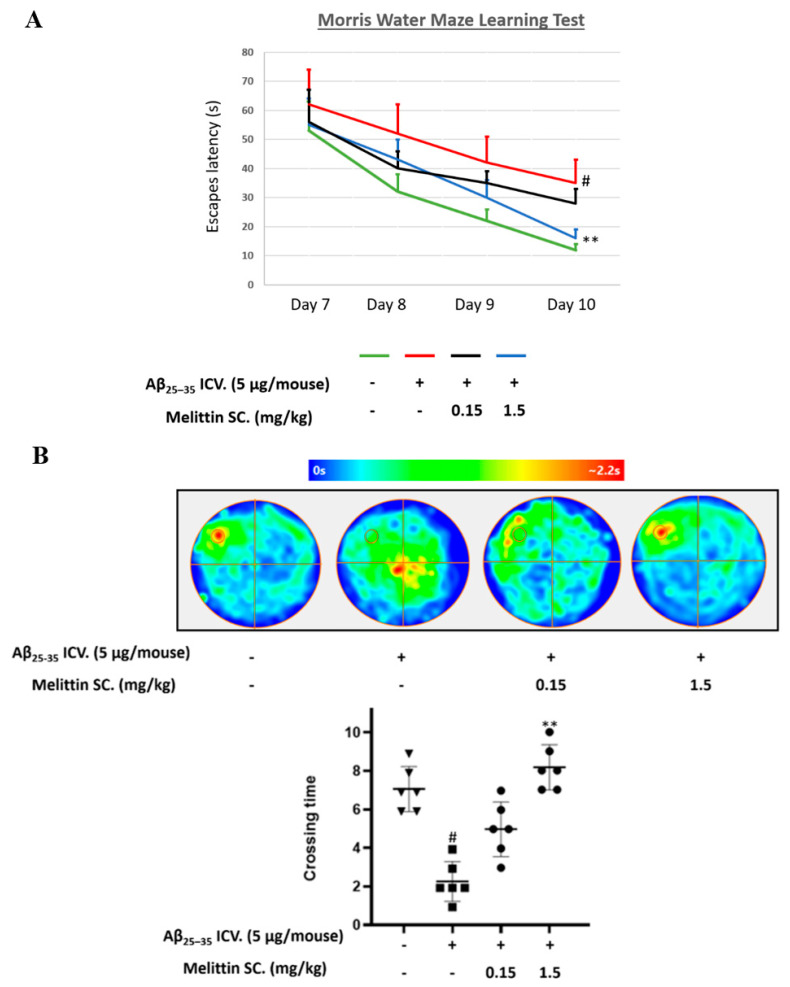
Protective effect of melittin on memory in Aβ_25–35_-treated mice studied in a Morris water maze experiment. Two tests (**A**,**B**) exhibited a decrease in learning and memory performance when mice were induced with Aβ_25–35_ ICV., and melittin 1.5 mg/kg significantly reversed this cognitive disfunction effect. The water maze behavior experiment recorded the escape latency time on days 7–10. (**A**) Animal escape latency performance on days 7 to 10. To conduct this experiment: Mice are administrated melittin (1.5 or 0.15 mg/kg SC.) after Aβ_25–35_ treatment (5 μg/mouse, ICV.), a training session was conducted on day 6, and the escape latency test was performed on days 7 to 10. (**B**) A probe test was carried out at the end of day 10 to further reinforce behavior evaluations. To conduct this experiment: The platform was removed, and mice were let to swim freely for 2 min to determine their memory of the disappeared platform location. The color scale indicates the average distribution position of animals within each group. The circle located in the upper-left quadrant represents the platform location. Data are presented as mean ± standard error values of the sextuple determinations. # *p* < 0.01 vs. control ** *p* < 0.01 vs. Aβ_25–35_ only-treated group.

To investigate the neuroprotective effect of melittin in an in vivo model, we used Aβ_25–35_ ICV. injections to induce a learning memory-deficit mouse model of neurodegenerative disease. We performed MWM sequentially for four days (days 7 to 10) and analyzed the alteration of melittin (0.15–1.5 mg/kg) on the escape latency time and the number of platform area crossings by mice. Melittin treatment (1.5 mg/kg) significantly reduced the escape latency of mice on the 10th day (Figure 6A) and the crossing time (Figure 6B) in the probe test compared to Aβ_25–35_ treatment only, implying the successful recovery of memory impairment by melittin in an animal model of cognitive dysfunction, a novel finding.

### 3.6. In Vivo Immunohistochemistry Study

**Figure 7:**
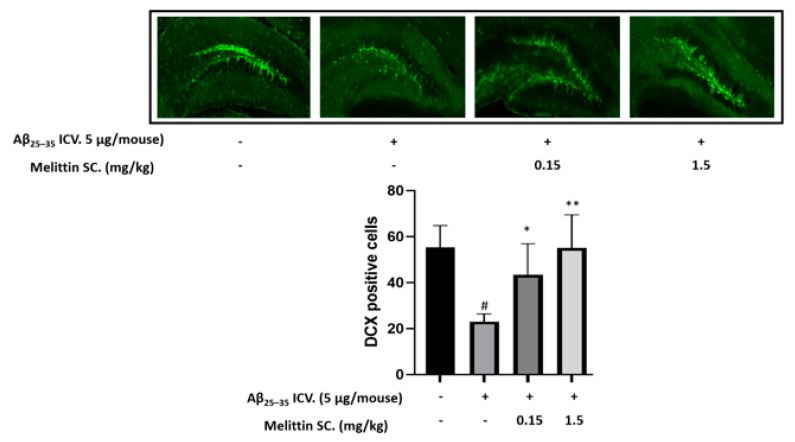
Protective effect of melittin on neuronal cells in the dentate gyrus region against Aβ_25–35_ induced stress. The result indicated that Aβ_25–35_ ICV. treatment reduced neuron cell neurogenesis significantly, and this was reversed by melittin. To conduct this experiment: Brain sections stained with doublecortin antibody, and the hippocampal dentate gyrus area were examined. Data are presented as the mean ± standard error of the sextuple determinations. # *p* < 0.01 vs. control * *p* < 0.05 and ** *p* < 0.01 vs. Aβ_25–35_ only-treated group.

We selected the dentate gyrus region to study the physiology of neuronal cells in the hippocampal region. The results indicated that Aβ_25–35_ ICV. injection decreased the number of doublecortin-positive cells in the dentate gyrus, whereas melittin SC. injection (1.5 mg/kg) increased it by 2.5-fold (Figure 7).

### 3.7. Oxidative and Inflammation Markers in Mouse Hippocampus and Serum

**Figure 8:**
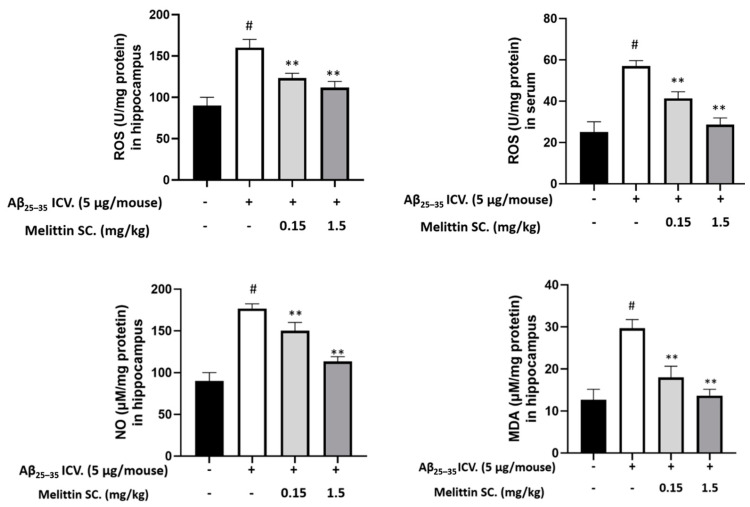
Major antioxidant parameters were examined in in vivo samples. The result showed that Aβ_25–35_ ICV. treatment significantly increased oxidative stress in the hippocampus and serum, which was reversed by melittin treatment. To conduct this experiment: Hippocampus tissue and serum were prepared, and ROS, NO, and MDA levels were examined as described in the materials and methods section. Data are presented as the mean ± standard error of the sextuple determinations. # *p* < 0.01 vs. control ** *p* < 0.01 vs. Aβ_25–35_ only treated group.

Aβ_25–35_ significantly increased ROS levels (2-fold) in both hippocampal tissue and mouse serum. Melittin ameliorated ROS levels in a dose-dependent manner. In addition, NO and MDA levels were significantly reduced in the melittin-treated groups (Figure 8). This is consistent with the previous results showing that melittin improved oxidative stress induced by Aβ_25–35_ in vitro. 

### 3.8. Study of In Vivo Key Marker Proteins

Histochemistry exhibited melittin’s capacity to enhance mouse hippocampal neurogenesis. We further confirm this via the expression of BNDF and p-CREB (Figure 7) in the hippocampus. The results suggested that BNDF and p-CREB were depleted in the Aβ_25–35_ only-treated group but were significantly upregulated in the melittin-treated groups (Figure 8).

**Figure 9:**
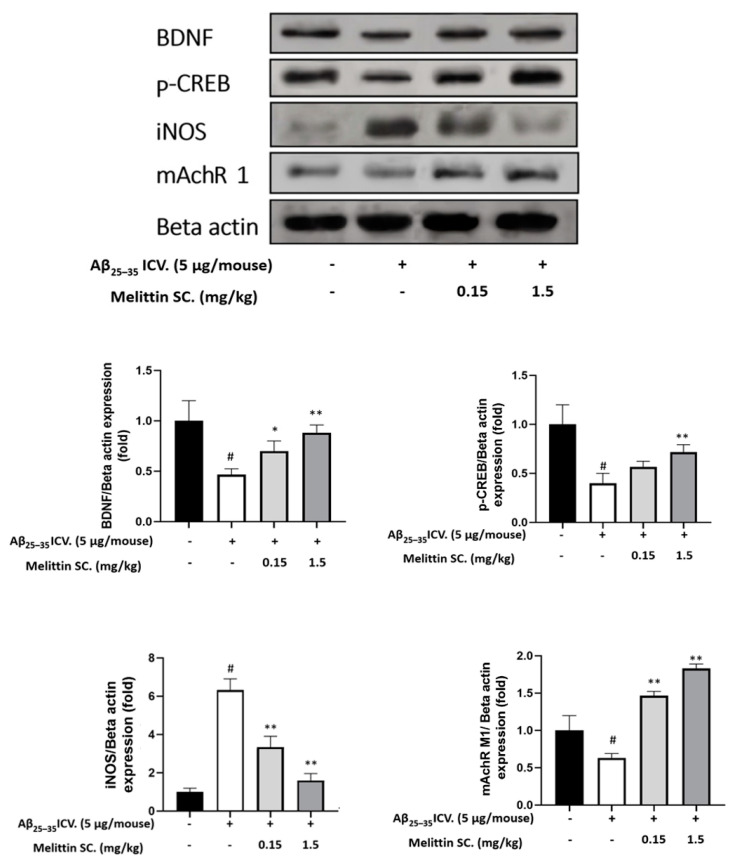
Levels of BDNF, p-CREB, iNOS, and mAchR1 protein expression in the mouse hippocampus show the dose-dependent protective effect of melittin against Aβ_25–35_-induced neuro-imbalance in vivo. To conduct this experiment: Western blot experiment was performed on protein extracts obtained from hippocampus tissue as described in the materials and methods section. Data are presented as the mean ± standard error values of the sextuple determinations. # *p* < 0.01 vs. control * *p* < 0.05 and ** *p* < 0.01 vs. Aβ_25–35_ only-treated group.

The hippocampal NO concentration was previously shown to be reduced by melittin (Figure 9); we reconfirmed this by the reduction of iNOS expression by melittin. As inflammation plays a decisive role in neurodegeneration, which can be induced by Aβ_25–35_ [35], the ability of melittin to normalize iNOS protein overexpression further confirms the effect of this candidate drug on suppressing dementia development in an in vivo model.

### 3.9. Study on In Vivo Neurotransmitter Alterations

**Figure 10:**
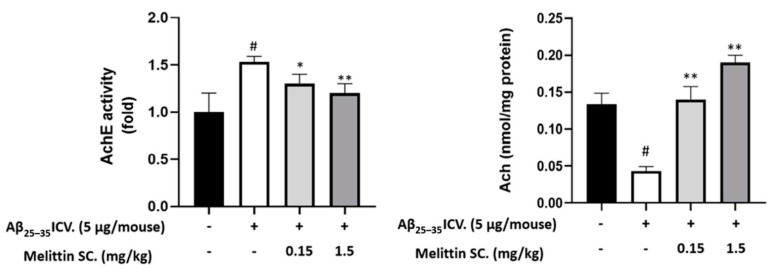
Levels of AchE activity and Ach content in the mouse brain. The cholinergic system exhibited ameliorations as melittin was administrated, against Aβ_25–35_ ICV.-induced neurodegeneration. To conduct this experiment: Analysis were performed on hippocampal tissue as described in the materials and methods section. Data are presented as the mean ± standard error of the sextuple determinations. # *p* < 0.01 vs. control * *p* < 0.05 and ** *p* < 0.01 vs. Aβ_25–35_ only-treated group.

In neurodegeneration stages, studies have shown a clear reduction in neurotransmitters such as acetylcholine and neuroreceptors such as M1 muscarinic acetylcholine receptors (mAchR 1) [36] and an over-increase in acetylcholinesterase [37]. In our experiment, in hippocampal samples, these mentioned cholinergic parameters were disrupted by Aβ_25–35_ ICV. injection. However, they were normalized by melittin, which further consolidates the effect of this compound in an in vivo neurodegenerative model (Figure 9—mAChR 1 protein expression & Figure 10).

## 4. Discussion

Neurodegenerative diseases, including Alzheimer which results in a decrease of human cognitive function, have become the main obstacles that degrade human life span and the overall wellbeing [38].

Through aging, Aβ is a product that naturally emerges and accumulates in the brain. Accumulated Aβ gradually forms aggregates with beta-sheet structures and has the capacity to alter the intercellular redox balance of neurons cells. This is considered a major cause of the onset of cognitive dysfunction [12,13,39]. Among Aβ variances, the Aβ_25–35_ fragment is the shortest segment of full-length Aβ_1_–_42_that can form a beta-sheet; this fragment was proven to have similar oxidative neurotoxicity output compared with the full-length Aβ_1_–_42_ [14]. With good solubility and stress-induced efficiency, interest in this undecapeptide Aβ_25–35_ has grown over the last decade [15].

In detail, both full-length and short Aβs were proven to form ion-like channels in cell membranes that promote Ca^2+^ influx, destabilize intercellular balance, and produce cellular oxidative stress [40,41,42]. Another source of oxidative stress arises as Aβ_25–35_ and Aβ_1_–_42_were also both found to cause mitochondrial abnormalities via the deactivation of mitochondrial complex IV [43,44,45,46]. In neurodegenerative progression, ROS overproduction from these important events causes damage to the cellular structure, increases MDA, LDH, and protein carbonyl levels, eventually initiates neuron cell death mechanisms, and promotes neuro-cognitive impairments [10,47,48]. Explaining this similar effect of Aβ_25_–_35_ and Aβ_1-42_ is still a topic of debate, but the single methionine-35 located in both Aβs was mentioned as its redox states are significantly important for Aβ-correlated free radical oxidative stress and neurotoxicity causes [45,49,50,51].

In the in vitro study, our results demonstrated that Aβ_25–35_ induced injury in HT-22 cells, including massive ROS release, resulting in MDA and LDH leakage and increased protein carbonyl levels, which substantially reduced HT-22 cell viability. Melittin dramatically mitigated Aβ_25–35_-induced oxidative stress injury by reducing ROS, MDA, SOD, and eventually, the protein carbonyl levels. To further explain these phenomena, we examined the melittin effect on the generation of HO-1, an important component of the cellular antioxidant system. This protein expression is inducible and has been demonstrated to shield cells against oxidative damage [16]. HO-1 expression is regulated by Nrf2. In the primitive stage, Nrf2 is retained in the Nrf2-Keap1 complex, and activation of cellular protection mechanisms leads to the separation of Nrf2 from the complex and transfer to the nucleus. Nrf2 acts as a transcription factor in the antioxidant response element (ARE) gene region, which in turn upregulates the expression of the HO-1 gene [10,33]. Our results showed that under cellular stress-induced conditions, melittin markedly upregulated the nuclear translocation of Nrf2 and enhanced the overall production of antioxidant enzyme activity, suggesting that the beneficial effect of melittin on Aβ_25–35_-induced HT22 cell injury was attributed to its antioxidant properties. This is in line with the findings of previous studies indicating that melittin enhances Nrf2 nuclear translocation and subsequently upregulates the expression of important antioxidant genes such as HO-1 [8,9].

One other mechanism of melittin can be related to the TrkB/CREB/BDNF pathway, which is a commonly studied direction along with antioxidant aspects [11,31,52,53]. The activation of TrkB can lead to downstream enhancement of both cellular antioxidant defensive and brain-derived neurotrophic factor neuro-proliferative shields [10]. In our experiment, Aβ_25–35_ presence significantly depleted the TrkB/CREB/BDNF pathway. Remarkably, melittin induced p-TrkB activation, increased the amount of p-CREB transcription factor, and in turn upregulated the expression of BDNF [54]. Moreover, BDNF was examined to stimulate subsets of Trk receptors and can further protect neuronal cells from oxidative stress-induced cell death [36,55]. The grounds above suggested the mechanism on how melittin demonstrated the ability to protect neuron cell HT22 apoptosis induced by Aβ_25–35_, an protective effect which was confirmed by the normalization of Bax/Bcl-2 ration, apoptosis-inducing factor, Calpain, CytoC, and CleaCas3.

For in vivo research, the injection of Aβ_25–35_ into mice brains was a utilized as a mean to induce oxidative stress, initiating synaptic loss, suppressing neurogenesis, and resulting in cognitive impairments in animal experiments [15,20,21,22]. This model elicits a considerable degree of Alzheimer’s progression signs and neurodegeneration conditions in general [21].

In this study, melittin significantly enhanced the memory and learning abilities of cognitive impairment-induced animals, a novel finding. The neuroprocessing ability of mice is closely related to hippocampal physiology [56]. Among the many anatomical parts of the hippocampus, the dentate gyrus is commonly studied and plays a key role in the formation, recall, and discrimination of episodic memory [57]. The significant increase in neurogenesis in this region has proven the effect of melittin at an anatomical scale.

To further explore the antioxidant property of melittin, we measured ROS and MDA levels in the hippocampus and serum; the levels of these parameters were significantly increase due to Aβ_25–35_ ICV., and melittin treatment clearly decreased these oxidative stress markers. Our results showed melittin reduced the amount of NO accumulation and iNOS protein expression in the hippocampus, which indicates melittin’s effect in lowering neuronal-derived nitric oxide—a key element stimulating neural diseases [58,59].

Maintenance of the balance of the cholinergic system is necessary for normal memory function [30]. Previous studies have revealed that patients with Alzheimer’s disease have downregulated expression of mAChR 1; hence, it is an important neuroreceptor to study in the cholinergic system [30]. In the synaptic cleft, ACh binds to postsynaptic mAChRs, and the synaptic signal communicates sequentially with the cyclic adenosine monophosphate/protein kinase A/CREB signaling pathway via G-coupled protein receptors. This bridge reflects the reality that the cholinergic signaling system and intercellular neurotrophic factors are two reciprocal entities. The disruption of one can cause a negative effect on the other and they both synergistically facilitate the grounds for neuronal grow and brain normal cognitive functions [60]. In our study, downregulation of mAChR 1 expression by Aβ_25–35_ in hippocampal tissues was significantly normalized by melittin pretreatment. Excessive AChE activity leads to a decrease in the Ach level in hippocampal cholinergic synapses. Aβ_25–35_ ICV. injection augmented AChE activity by 1.5-fold in hippocampal tissue, whereas pretreatment with melittin completely attenuated the excessive activation of AChE and increased ACh levels. Regarding intercellular neurotrophic factors, the transcription factor CREB, which plays a key role in BDNF synthesis, is essential for memory and synaptic plasticity [61]. In line with the in vitro experiments, our in vivo results showed that melittin-treated mice also had increased hippocampal p-CREB and BDNF levels.

Therefore, through both in vivo and in vitro experiments, melittin had proved itself to be a drug candidate combating neurovegetative disease.

The drug administration route of melittin is often subcutaneous, which can cause adverse effects if over-dosed [62]. Although melittin exhibited the ability to recover cognitive function in neurodegenerative-induced models, its irritation properties should be alleviated, and an optimal dosage for humans should be determined. In other disease research with melittin, up-to-date recombinant technology and computational bioinformatics modified the specific amino acid sequences and created a specialized-engineered-melittin. This produced effective augmentation and enhanced drug delivery, which enabled melittin to event be intravenously injected and to target a specific group of malarian cells [63]. Such advances can alleviate the side effects of melittin and further increase the popularity of melittin treatment.

## 5. Conclusions

Melittin is the dominant component of four most common honeybee species’ venoms. The present study demonstrates that melittin exerts neuroprotective effects by regulating the activation of Nrf2/HO-1 and the TrkB/CREB/BDNF pathways in HT22 cells. Also, for the first time to our knowledge, melittin was found to recover the depleted learning and memory ability in a neurodegenerative in vivo model. These findings contribute to clarify the underlying ability and mechanism of melittin to protect neuronal cells from neuronal-oxidative stress and promote the role of melittin as a medication combating cognitive impairment disorders.

## Data Availability

The data presented in this study are available in article.

## Abbreviations

ROS, reactive oxygen species; BDNF, brain-derived neurotrophic factor; TrkB, tropomyosin-related kinase receptor B; Nrf2, nuclear factor erythroid 2-like 2; HO-1, heme oxygenase-1; MWM, Morris water maze; SOD, Superoxide Dismutase; MDA, Lipid Peroxidation; LDH, Lactate Dehydrogenase; AchE, acetylcholine esterase; Ach, acetylcholine; CytoC, Cytochrome c; CleaCas3, Cleaved Caspase-3; NO, nitric oxide; iNOS, inducible nitric oxide synthase; CREB, cAMP Response Element-Binding; mAchR 1, M1 muscarinic acetylcholine receptors; SC., Subcutaneous Injection; ICV., Intracerebroventricular injection; Aβ, beta amyloid. AIF, Apoptosis-inducing factor; DAPI, 4′,6-diamidino-2-phenylindole; RFP, Red Fluorescent Protein; GFP, Green Fluorescent Protein; DCFDA, 2’,7’-dichlorodihydrofluorescein diacetate; p-, phosphorylated-.
